# Single center experience with more than 600 drug desensitization in Colombia

**DOI:** 10.3389/falgy.2024.1460326

**Published:** 2024-08-30

**Authors:** Verónica Pardo-Manrique, Luis Fernando Ramírez-Zuluaga, Diana Lucia Silva-Espinosa, Leidy Johanna Hurtado-Bermudez, Inés Elvira Gómez-Hernández, Manuela Olaya-Hernández, Carlos Daniel Serrano-Reyes

**Affiliations:** ^1^ Centro de Investigaciones Clínicas, Fundación Valle del Lili, Cali, Colombia; ^2^ Facultad de Ciencias de la Salud, Universidad Icesi, Cali, Colombia; ^3^ Servicio de Alergología, Fundación Valle del Lili, Cali, Colombia

**Keywords:** hypersensitivity, desensitization, chemotherapeutics, monoclonal antibodies, antibiotics, breakthrough reactions

## Abstract

**Background:**

Drug hypersensitivity reactions (DHRs) have a significant impact on both, patient and their treating physicians; it is considered a public health concern. The history of allergy to drugs, limits therapeutic options and will lead to the use of more expensive and potentially less effective options. Drug desensitization (DD) is considered as a procedure with a positive impact on the prognosis of the patient's disease. The objective of this study is to describe the experience with a substantial number of drugs desensitization in a fourth level center in Cali, Colombia.

**Methods:**

An observational, cross-sectional and descriptive study was conducted. Patients with DHRs who underwent a standardized institutional DD protocol, between March of 2012 and May of 2023, were included.

**Results:**

Two hundred forty-one patients were included. The median age was 47.8 years (4–88). One hundred fifty-six (64.7%) were women, including three who were pregnant. A total of 641 DDs were performed. The most frequent groups of drugs for which the desensitization was performed were monoclonal antibodies in 83 patients (34.4%), chemotherapeutic agents in 53 (21.6%), NSAIDs in 44 (18.2%), and antibiotics in 42 (17.4%). Eighty-seven patients (36.1%) experienced hypersensitivity to the culprit drug on first exposure, while 154 (63.9%) exhibited reactions during subsequent cycles. The main clinical presentation that gave rise to desensitization was anaphylaxis in 125 patients (51.8%), followed by cutaneous symptoms in 106 patients (44%). The predominant observed endophenotype was type 1 in 188 patients (78.3%), followed by mixed type in 46 patients (19.2%). Breakthrough reactions were observed in 50 patients (20.7%). Tolerance to DD was achieved in 636 of the procedures (99.2%), allowing the continuity of treatment of choice for the underlying disease.

**Conclusions:**

Most desensitized patients were women with type I reactions. Monoclonal antibodies were the most frequent culprit drugs. DD in patients with DHRs is a useful, safe and effective procedure. The administration of the implicated drug had a positive impact on the course of the disease in these patients.

## 1 Introduction

Adverse drug reactions (ADRs) are a public health issue that increase hospital admissions, length of stay, and mortality (1). The World Health Organization (WHO) considers ADRs to be one of the top 10 causes of death worldwide (2). Hypersensitivity reactions (HSRs) represent 15%–20% of ADRs, affecting 7% of the general population and 10%–20% of hospitalized patients (3, 4). The real morbidity caused by HSRs is difficult to determine due to the limited quality of available epidemiological data, as most published studies either do not specify the type of ADR or the allergy diagnosis is not confirmed and is simply based on patient-reported labels (5–7).

In Europe, drug-induced HSRs represents for 1%–2% of hospital admissions (5, 8) and 14% of emergency visits (0.6% due to anaphylaxis), resulting in hospitalization in 15% of cases (9). In the pediatric population, the incidence of HSRs varies widely, from 0.6% to 16.8% among hospitalized patients, significantly contributing to emergency consultations and hospitalizations (5, 9). Specific data for Latin America are limited. A 2014 study included 868 drug-induced HSRs and revealed a higher frequency in females, a predominance of cutaneous manifestations, and frequent involvement of non-steroidal anti-inflammatory drugs (NSAIDs) and beta-lactam antibiotics (6).

HSRs are associated with negative patient outcomes, such as prolonged hospital stays, the need for invasive interventions, and increased morbidity and mortality, especially when the implicated drugs are essential and lack suitable alternatives, such as biological therapies, chemotherapeutic agents, antibiotics, antituberculous drugs, and acetylsalicylic acid (ASA), among others (3). In these cases, patients face receiving a less effective drug for their disease, increasing morbidity and mortality.

In response, rapid drug desensitization (RDD) has emerged as the strategy of choice to allow the safe administration of indispensable medications in patients with a history of HSRs. RDD aims to induce temporary tolerance by starting with very low doses and gradually increasing them until the full therapeutic dose is reached; once this occurs, RDD is considered successful (10–12). This technique not only reduces associated morbidity and mortality but also minimizes adverse effects and improves patient quality of life (10, 13).

Several mechanisms for RDD have been proposed, though they are not fully understood and may complement each other. In type I HSR, increased internalization of the IgE/FcεRI complex occurs due to enhanced cross-linking at low antigen concentrations (10). During desensitization, these complexes are initially internalized, while the remaining antigen-loaded IgE remains on the FcεRI alpha chain at the membrane level (14). Inhibitory receptors, such as gp49B1 a transmembrane glycoprotein with two immunoreceptor tyrosine-based inhibitory motifs (ITIMs) on mouse bone marrow mast cells (mBMMCs) are believed to play a role in desensitization. These ITIMs bind to SH2-containing protein tyrosine phosphatases like SHP-1, SHP-2, and SHIP-1, which can dephosphorylate Syk and other early signal transduction molecules, shifting the signal towards an inhibitory pathway (10).

Studies in Europe, Asia, and the United States have documented the safety and efficacy of desensitization to various drugs, especially chemotherapeutic agents and monoclonal antibodies (mAbs) (13, 15–19). A Korean study reported a high success rate (99%) after performing 1,143 desensitizations in 228 patients, most of which were to platinum agents, taxanes, and mAbs (15). Similarly, a recent systematic review highlighted the effectiveness of RDD for taxanes, with success rates of 95%–100% using standardized protocols (20). In Colombia, a study was published that included 14 patients in which 45 desensitizations to chemotherapeutics and mAbs were performed, being successful in all cases (1).

Available literature supports the safety of desensitization, although it recognizes inherent risks, thus it should be performed in an appropriate medical setting and by qualified personnel (9, 21). Acknowledging the importance of desensitization in clinical practice and the scarcity of evidence in our country and in Latin America, this study aimed to describe the sociodemographic, clinical characteristics, and outcomes of patients with drug-induced HSRs who underwent a desensitization protocol.

## 2 Methodology

This was an observational, cross-sectional study that included patients with a history of drug hypersensitivity who underwent at least one desensitization protocol at the Fundación Valle de Lili, between March 2012 and May 2023. Information was collected from medical record reviews. Sociodemographic and clinical variables were included, including the reaction phenotype and the success or failure of the procedure. The study was approved by the Institutional Ethics Committee of the Fundación Valle de Lili and adhered to the principles of the Declaration of Helsinki.

### 2.1 Statistical analysis

For univariate analysis, measures of central tendency and dispersion were used for quantitative variables, and frequencies and percentages for categorical variables. The standard criterion for quantitative variables was determined with the Kolmogorov-Smirnov test; in the case of normality, means with standard deviation were obtained, and in the absence of normality, medians with interquartile range were used.

## 3 Results

A total of 241 patients were included. One hundred fifty-six (64.7%) were women; the median age was 47.8 years. The most frequent comorbidities were autoimmune disease in 91 patients (37.7%), neoplasms, both solid and hematologic, in 83 (34.4%), and arterial hypertension in 67 (28.3%) (Table 1).

**Table 1:** Clinical and sociodemographic characteristics of patients undergoing desensitization.

Variable	*n* = 241 (%)
Demographics	
Female	156 (64.7)
Age in years, Mean (SD)	47.8 (20.1)
Intensity of the initial allergic reaction	
Mild (local involvement of a single system)	92 (38.2)
Moderate (generalized involvement of one system or two or more systems)	124 (51.4)
Severe (presence of hypoxia, hypotension, or neurological involvement)	25 (10.4)
Symptoms of the allergic reaction	
Anaphylaxis	125 (51.8)
Cutaneous symptoms	104 (43.1)
Respiratory symptoms	12 (4.9)
Endophenotype	
Type 1	188 (78.3)
Mixed	46 (19.2)
Cytokine release reaction	6 (2.5)
Route of drug administration	
Intravenous	156 (64.7)
Oral	81 (33.6)
Subcutaneous	4 (1.7)
Latency time of the reaction	
Between 1 and 6 h	161 (66.8)
Less than 1 h	67 (27.8)
More than 6 h	13 (5.4)
Timing of the reaction occurrence	
With the first drug administration	87 (36.1)
With the second drug administration	77 (31.9)
With the third drug administration	36 (14.9)
After the fourth or more drug administrations	41 (17.0)
Tryptase	
No	241 (100)
Skin tests	
Yes	10 (4.1)
History of drug hypersensitivity	
Yes	69 (28.6)
Comorbidities	
Autoimmune disease	91 (37.7)
Neoplasms	83 (34.4)
Hypertension	67 (28.3)
Infections	49 (20.3)
Cardiovascular disease	45 (18.7)
Hypothyroidism	32 (13.3)
Pulmonary disease	29 (12.0)
Diabetes mellitus	24 (10.2)
Renal disease	22 (9.3)
Psychiatric disease	17 (7.1)
Immunodeficiency	14 (6.3)
Asthma	12 (5.0)
Allergic rhinitis	10 (4.2)
Chronic urticaria	7 (2.9)
Dermatitis	4 (1.7)
Conjunctivitis	2 (0.8)

SD, standard deviation.

Anaphylaxis was the most common initial reaction, occurring in 125 patients (51.8%), followed by cutaneous manifestations in 106 (44%). In 87 patients (36.1%), HSRs occurred with the first administration of the drug, while in 154 (63.9%), they occurred during subsequent administrations. The latency period after exposure was less than one hour in 67 patients (27.8%), between one and six hours in 161 (66.8%), and more than six hours in 13 (5.39%). The most common endophenotype was type I in 188 patients (78.3%), followed by mixed in 46 (19.3%) (Table 1).

The administration of the drug was intravenously in 156 patients (64.7%), orally in 81 (33.6%), and subcutaneously in 4 (1.7%). The frequency of desensitizations according to the drug group was: mAbs in 83 patients (34.4%), chemotherapeutic agents in 53 (21.6%), NSAIDs in 44 (18.2%), and antibiotics in 42 (17%). Regarding the specific drug, desensitizations were most frequently performed with rituximab in 78 patients (32.37%), oxaliplatin in 15 (6.22%), ASA in 44 (18.26%), and trimethoprim- sulfamethoxazole in 27 (11.20%) (Table 2).

**Table 2:** Medications for which desensitization protocol was used.

Type of Medication	No. Patients (%)	No. Rapid Drug Desensitizations (%)
Monoclonal Antibody	**83** (**34.4)**	**333** (**51.9)**
Rituximab	78 (32.37)	299 (46.6)
Canakinumab	1 (0.41)	7 (1.09)
Daratumumab	1 (0.41)	2 (0.3)
Infliximab	1 (0.41)	1 (0.15)
Pertuzumab	1 (0.41)	8 (1.2)
Tocilizumab	1 (0.41)	16 (2.5)
Chemotherapeutic Agent	**53** (**21.9)**	**140** (**21.8)**
Oxaliplatin	15 (6.22)	38 (5.9)
Paclitaxel	14 (5.81)	49 (7.6)
Carboplatin	6 (2.49)	15 (2.3)
L-asparaginase	5 (2.7)	8 (1.2)
Cytarabine	3 (1.24)	4 (0.6)
Peg-asparaginase	2 (0.83)	3 (0.46)
Cyclophosphamide	1 (0.41)	9 (1.4)
Cisplatin	1 (0.41)	2 (0.3)
Docetaxel	1 (0.41)	3 (0.46)
Liposomal doxorubicin	1 (0.41)	1 (0.15)
Gemcitabine	1 (0.41)	1 (0.15)
Irinotecan	1 (0.41)	5 (0.78)
Lenalidomide	1 (0.41)	1 (0.15)
Methotrexate	1 (0.41)	1 (0.15)
NSAIDs	**44** (**18.2)**	**43** (**6.7)**
ASA	44 (18.2)	43 (6.7)
Antibiotic	**42** (**17.0)**	**51** (**7.9)**
Trimethoprim-sulfamethoxazole	27 (11.20)	33 (5.1)
Benzathine penicillin	3 (1.24)	3 (0.46)
Meropenem	2 (0.83)	5 (0.78)
Amoxicillin	1 (0.41)	0
Ampicillin	1 (0.41)	2 (0.3)
Ampicillin-sulbactam	1 (0.41)	1 (0.15)
Amphotericin B	1 (0.41)	1 (0.15)
Ceftriaxone	1 (0.41)	1 (0.15)
Clindamycin	1 (0.41)	1 (0.15)
Levofloxacin	1 (0.41)	1 (0.15)
Piperacillin-tazobactam	1 (0.41)	1 (0.15)
Rifampicin	1 (0.41)	1 (0.15)
Vancomycin	1 (0.41)	1 (0.15)
Other	**19** (**7.8)**	**74** (**11.5)**
Iron sucrose	4 (1.66)	20 (3.1)
Immunoglobulin G	3 (1.24)	26 (4.05)
Elosulfase alfa	2 (0.83)	15 (2.3)
Etravirine	1 (0.41)	1 (0.15)
Feiba	1 (0.41)	1 (0.15)
Furosemide	1 (0.41)	1 (0.15)
Levothyroxine	1 (0.41)	1 (0.15)
Methadone	1 (0.41)	1 (0.15)
Rosuvastatin	1 (0.41)	1 (0.15)
Somatostatin	1 (0.41)	1 (0.15)
Ferrous sulfate	1 (0.41)	0
Triptorelin	1 (0.41)	5 (0.78)
Warfarin	1 (0.41)	1 (0.15)

The values ​​in bold are the total number and % in each of the drug groups.

During the study period, a total of 641 desensitizations were performed, with an average of 2.74 desensitizations per patient (Range 1–24). It was identified that 10 patients required more than 10 desensitizations.

Regarding clinical outcomes, it was observed that 50 patients (20.7%) experienced HSRs during the desensitization protocol. Of these, 31 patients (62%) had mild HSRs, and 19 (38%) had moderate to severe HSRs. These reactions were managed with temporary suspension of the protocol and administration of systemic antihistamines and corticosteroids in 34 patients (75.5%), while adrenaline was required in only 4 (8.8%). The administration of the full dose of the implicated drug was achieved in 636 desensitizations (99.2%) (Table 3).

**Table 3:** Description of desensitizations performed.

Desensitization	*n* = (%)
Success of desensitization protocol	636 (99.2)
Number of desensitizations	
One	133 (55.2)
Two	36 (14.9)
Three or more	72 (29.9)
Type of protocol used	
Intravenous (3 bags - 12 steps)	473 (73.7)
Intravenous (4 bags - 16 steps)	2 (0.3)
Intravenous (variable number of steps and/or bags)	71 (11)
Oral (5–8 steps)	82 (12.7)
Subcutaneous	13 (2)
Premedication received before protocol	
Yes	169 (70.1)
No	72 (29.9)
Clinical outcomes	
Allergic reactions during protocol	50 (20.7)
Reactions after desensitization protocol^a^	2 (0.8)
Hospitalization	1 (0.4)
Rescue treatment during protocol	
Antihistamines	34 (75.5)
Corticosteroids	34 (75.5)
Analgesic	5 (11.1)
Epinephrine	4 (8.8)
Oxygen	4 (8.8)
Beta-2 agonist	3 (6.6)

^a^ Two patients developed maculopapular rash in the days following desensitization.

Of the 50 patients who experienced reactions, 39 (78%) occurred during the first desensitization, 9 (18%) during the second, one (2%) during the sixth and one (2%) during the ninth desensitization. Bivariate analysis identified a significant association between the presence of reactions and being under 50 years of age (*p* = 0.010). No other relevant associations were found.

## 4 Discussion

Drug desensitization is a highly impactful intervention, as it allows patients to receive essential drugs for managing their underlying disease. This study analyzed the clinical characteristics of 641 desensitization procedures in 241 patients. In line with previous studies, the most patients were middle- aged women (15, 17, 22), suggesting a relationship with the higher prevalence of autoimmune diseases and gynecological neoplasms (13, 15).

In 161 patients (66.8%), initial reactions occurred between the first and sixth hours after exposure to the implicated drug, while in 67 (27.8%), they occurred in less than one hour; 36.1% occurred with the first exposure and 63.9% in subsequent exposures. The most common endophenotype was type I, presented in 188 patients (78.3%), data that are consistent with a previous study that included 79 patients and 267 RDDs 267 (22). It should be noted that type I reactions typically require prior sensitization, so they tend to occur after repeated exposures (23). The symptoms of these can be like those of cytokine release syndrome, with the difference that the latter can occur without prior exposure to the drug.

In this study, the classification of endophenotypes for HSR was carried out considering the type of drug, previous exposure, latency time, and clinical characteristics observed during the index reaction. The first endotype, known as endotype I, is characterized by a range of immediate symptoms including naso-ocular manifestations, cardiovascular involvement (e.g., hypotension, tachycardia), lower airway symptoms (e.g., hypoxia, wheezing, bronchoconstriction), gastrointestinal symptoms (e.g., vomiting, cramps, diarrhea), and cutaneous symptoms (e.g., urticaria, angioedema, pruritus, flushing). This clinical profile is commonly associated with IgE-mediated reactions. The second endotype, cytokine release endotype, is distinguished by constitutional symptoms (e.g., fever, chills, rigors, headache), cardiovascular involvement (e.g., tachycardia, hypertension or hypotension), and truncal and limb musculoskeletal pain, suggesting a cytokine-mediated response rather than IgE-mediated. The mixed endotype includes the simultaneous presence of symptoms from both previously described endotypes.

Of note, this cohort did not include patients with delayed hypersensitivity reactions such as fixed drug erythema or drug-induced exanthema.

Most HSRs to rituximab, the drug most implicated in this study, occurred during the first treatment cycle, which is consistent with literature reports (16). It has been documented that up to 50% of reactions to this drug occur during the first exposure, supporting the existence of the cytokine release endophenotype (24).

Regarding chemotherapeutic agents, previous studies show that HSRs tend to occur after 6 to 10 exposures with platinum agents and after two exposures with taxanes (13, 17, 18, 25, 26). In this study, HSRs occurred after the fourth, and between the first and second exposures, respectively, which is similar to what has been previously described.

The initial reactions in this study were anaphylactic in most cases (51.8%), which also aligns with previous reports (2, 19). Additionally, a higher frequency of cutaneous and respiratory manifestations was observed, both as part of anaphylaxis and in an isolated form. Bavbek et al. (16) found a higher frequency of respiratory and cardiovascular symptoms in patients exposed to biological agents.

In none of the patients were biomarkers such as serum tryptase or interleukin-6 (IL-6) determined. The measurement of biomarkers is important in the endotyping of reactions, as elevated tryptase levels during these are associated with IgE-mediated endotypes, while high IL-6 values are related to cytokine release (23). Skin tests were performed in only 10 patients, yielding negative results. The purpose of these tests is to determine the phenotype, stratify risk, and guide treatment (10), but their implementation is obstructed by the high cost of some medications in the national context, which poses a significant challenge in clinical decision-making.

As in other series, mAbs and chemotherapeutic agents were the drugs most frequently implicated in HSRs (9, 15, 19). Specifically, rituximab and oxaliplatin topped the list, consistent with previous descriptions (16, 18, 27). However, unlike previous reports, where rituximab was primarily prescribed for hematologic neoplasms (16), in this study, it was for autoimmune diseases.

HSRs are more frequently described in patients with ovarian and breast neoplasms (18), possibly related to the indication of platinum agents and taxanes in these patients. In this study, although these neoplasms topped the frequency list, gastrointestinal tract neoplasms also occupied a relevant place.

It is noteworthy that this experience has strengthened communication, education, and teamwork with other specialties, such as rheumatology, oncology, and hematology, who have become aware that RDD allows the administration of the treatment of choice to patients who have experienced an HSR.

Regarding NSAID desensitizations, in this study, the indication for ASA was mainly related to the presence of coronary disease, and tolerance was successfully induced in all patients who required it. These results are consistent with those obtained by Rossini et al. (28), who achieved a 95.4% success rate with ASA desensitization in 330 patients with stable or suspected coronary disease.

In relation to antibiotics, trimethoprim-sulfamethoxazole (TMP-SMX) was the most implicated in HSRs, requiring a desensitization protocol in 27 patients (11.2%). The indication for all cases was prophylaxis for *Pneumocystis jirovecii* infection in immunosuppressed patients, and it was tolerated by all of them (29).

There were three pregnant women diagnosed with syphilis and allergic to penicillin who underwent desensitization with crystalline penicillin without complications. Recently, a case was reported in Chile involving two pregnant women who were successfully desensitized for the same indication with adequate tolerance (30).

Of the 641 RDDs reported in this study, more than 50% were performed intravenously. In this context, Lee et al. (31) reported successful desensitization to carboplatin in ten patients using a twelve-step and three solutions with varying drug concentrations protocol. In 2008, Castells et al. (17) published a series of 98 patients who underwent 413 desensitizations using a similar protocol. For several years, the institution has implemented a desensitization protocol by this route, with a dosage calculation method based on the exact prescribed dose for the patient and considering the specific pharmacological characteristics of the drug, especially about concentrations and maximum infusion rates described in the technical data sheet of each drug, thus avoiding drug wastage. As in previous reports (1, 13, 16, 17, 32, 33), this approach also involves the use of 3 different dilutions and 12 steps and can be adjusted individually according to the drug and the specific needs of each case. The 3-bag and 12-step, and 4-bag and 16-step protocols have become the most commonly used in clinical practice, validated by more than 3,000 scientific publications and proven to be effective and safe for chemotherapeutic agents, mAbs, and antibiotics, even in severe HSRs graves (17, 32, 34, 35). In this research, 473 procedures were performed using 3-bag and 12-step, and 2 using four solutions and sixteen steps. Additionally, some desensitizations were performed using 3 bags with fewer steps, and others using a single bag in multiple steps according to risk stratification. Recently, a study was published that included 434 desensitizations using an 11-step protocol with a single dilution, achieving drug administration in 99.5% of cases; however, the incidence of reactions was 49% (19).

Oral RDDs performed in this study (82 in total) used protocols between 5 and 8 steps and were all successful.

A limitation of this research was the low utilization of diagnostic tests, a widely debated topic in the literature. There is significant controversy over the ability of skin tests to confirm the underlying mechanism and predict future HSRs. However, it is important to note that a negative or ambiguous result in these tests should not affect the decision to carry out desensitization, especially if the patient's history suggests immediate hypersensitivity to the drug in question (36–38).

In this study, premedication with 500 mg of acetaminophen, corticosteroids (100 mg of hydrocortisone), and antihistamines (10 mg of cetirizine) was administered in all intravenously performed desensitizations. However, in oral or subcutaneous procedures, premedication was not applied in all cases but only where medically indicated, in accordance with current recommendations (21).

HSRs during RDD tend to be less intense than the initial reaction (16, 21). In the largest series reported to date, which included 1,142 desensitizations, it was observed that 26% of patients experienced a reaction during desensitization. Despite this, 99% of the patients successfully completed the procedure (15). In this study, 50 patients (20.7%) experienced HSRs during one of the desensitizations, with rituximab implicated in 28 of them (56%). Reaction rates between 29% and 40% during desensitization with this drug have been reported (39–42). In this study, the rate was 12.7% (38 of 299 desensitizations). This difference may be explained by the consistent use of premedication regardless of the severity of the initial reaction. It is important to mention that these reactions were more frequent in patients under 50 years old (*p* = 0.001). This could be related to a higher prevalence of autoimmune diseases and gynecological neoplasms in this age group, conditions for which the most implicated drugs were indicated.

As in other studies (17, 18), in this study the reactions were more frequent during the first cycles, indicating that both the frequency and severity of these tend to decrease with a greater number of desensitizations.

Almost all desensitizations (99.2%) were successful in this study, with “success” defined as the complete administration of the prescribed dose for the patient. These results are consistent with those reported in other studies (13, 15, 17, 27). Only in 5 procedures was the complete dose of the drug not administered due to severe reactions during the procedure, despite the administration of corticosteroids and antihistamines and adjustments to the protocol. These cases involved three patients who required rituximab, cytarabine, and human immunoglobulin G.

In addition to the limited use of diagnostic tests, another important limitation of this study was its retrospective nature. Nevertheless, its main value lies in being the largest series published in Latin America, describing the demographic, clinical characteristics, and outcomes of desensitization with various drugs. It is also important to note that this procedure is not routinely performed in all institutions in the country, which limits the possibility of a multicenter report but also adds relevance and originality to the analysis.

## 5 Conclusion

The data presented in this study support the usefulness, efficacy, and safety of RDD in Colombian patients with HSRs. The possibility of administering the implicated drug had a positive impact on the course of the disease in these patients, improving clinical outcomes and their quality of life. These findings contribute to the current knowledge on RDD in patients with drug-induced HSRs in Latin America and constitute an important basis for more extensive reports that include a larger number of centers and countries.

## Data Availability

The original contributions presented in the study are included in the article/Supplementary Material, further inquiries can be directed to the corresponding author.
